# Haptoglobin polymorphisms in Latin American populations

**DOI:** 10.1038/s41598-020-70755-y

**Published:** 2020-08-13

**Authors:** Mikiko Soejima, Yoshiro Koda

**Affiliations:** Department of Forensic Medicine, Kurume University School of Medicine, Kurume, 830-0011 Japan

**Keywords:** Genetic variation, Genetic testing

## Abstract

Several genetic polymorphisms of the haptoglobin gene (*HP*) or haptoglobin-related gene (*HPR*) were reported to show a population-specific distribution and to be associated with not only serum haptoglobin (HP) but also cholesterol levels. For such association studies, it is important to know the distribution of polymorphisms or their haplotypes in the populations concerned. However, no comprehensive genetic studies have explored this in Latin Americans, and not every human variation or genotype is available in a database. In this study, we determined the genotypes of common *HP* (*HP*^*1*^ and *HP*^*2*^), *HP*^*del*^, rs5471, rs5472, and rs2000999 in several Latin American populations. Haplotypes of rs5472-common *HP*-rs2000999 polymorphisms were estimated*.* We did not encounter any *HP*^*del*^, and the frequencies of rs5471 A, rs5472 A, *HP*^*1*^, and rs2000999 G were higher than their counterpart alleles in studied populations. All of the alleles with higher frequency in the Latin Americans are associated with higher serum HP and lower cholesterol levels. Both A-1-G (probably *HP*^*1S*^) and G-1-G (probably *HP*^*1F*^) haplotypes were higher in Latin American populations than those in other geographic regions. In addition, the genetic influx from populations of other continents into Peruvians seems to be relatively lower than that into other Latin Americans.

## Introduction

Haptoglobin (HP) is a plasma glycoprotein and is highly expressed in the liver^1,2^. HP binds hemoglobin (Hb) with a very high affinity to prevent both iron loss and kidney damage due to the oxidative activity of Hb during intravascular hemolysis^3^. The HP gene (*HP*) has two codominant alleles of *HP*^*1*^ and *HP*^*2*^ in humans, and as a result, HP has three common phenotypes, HP1-1, HP2-1, and HP2-2^3^.

*HP* locates on the long arm of chromosome 16 (16q22.3) beside the haptoglobin-related gene (*HPR*) and consists of five (*HP*^*1*^) or seven (*HP*^*2*^) exons. It has been considered that *HP*^*2*^ is generated by a 1.7-kb intragenic duplication of exons 3 and 4 of *HP*^*1*^^4^. However, a recent study suggested that *HP*^*1*^ likely arose from several recurring deletions of duplicated exons of *HP*^*2*^ by imputation from SNP haplotypes^5^. In any event, *HP*^*1*^and *HP*^*2*^ are copy number variations (CNVs). HP1 further has two subtypes, variants that migrate faster (HP1F) and slower (HP1S) on starch gel due to two amino acids, positions 52 and 53 (Asp-Lys for 1F, Asn-Glu for 1S), in the duplicated region^1^. HP2 also is divided into three subtypes: 2FS (containing 1F plus 1S sequences), 2SS (1S plus 1S), 2FF (1F plus 1F). The phenotyping of 1F/S is not easy, and it is also difficult to examine the polymorphic sites responsible for 1F/S directly by fast and simple methods such as real-time PCR due to the complexity of the gene structure^1,5^. An “indirect” PCR restriction fragment length polymorphism (PCR–RFLP) method has been used to distinguish the SNP associated with the polymorphic sites responsible for 1F/S^6,7^. We previously analyzed the genetic variation of the *HP* gene including *1F/S* in Ghanaians, Europeans, and Chinese, and found that the A and G alleles of a promoter SNP at position -55 (rs5472) seemed to be almost completely linked with *HP*^*1F*^ and *HP*^*1S*^, respectively^8^.

In addition to common *HP* polymorphisms, several rare variants of the HP phenotypes have been reported^3^. One of them is a complete *HP* deletion allele (*HP*^*del*^) that has an approximately 28 kb deletion extending from the *HP* promoter region to intron 4 of *HPR*^9^. *HP*^*del*^ homozygotes are at risk for developing anaphylactic transfusion reactions if they produce antibodies (IgE) to HP^9,10^. This allele has been found only in East and Southeast Asian populations so far^8,9,11–19^.

Because HP is one of the acute phase proteins, its serum level increases in various clinical states such as infectious diseases, malignancy, autoimmune disease, and tissue necrosis. On the other hand, levels decrease during hemolysis, ineffective erythropoiesis, liver disease, and late pregnancy^3,20^. In addition, gender, age, smoking, plasma Hb levels, serum lipids, and some genetic factors were shown to be associated with circulating HP levels^21,22^. Serum HP concentrations are reported to be associated with the common *HP* genotypes and *HP*^*del*^^3,12,23^. A SNP located in *HPR* intron 2 (rs2000999), which was originally identified as one of the genetic determinants of serum total cholesterol by a genome-wide association study (GWAS)^24^, was thereafter reported to be associated with the serum HP level^12,21–23,25^. A promoter SNP at position − 61 (rs5471), a characteristic SNP of Africans, was identified as a causal polymorphism of HP 2-1 modified phenotypes due to a decreased amount of HP2 polypeptide relative to that of HP1 polypeptide^6^. Recently we suggested that this SNP is a strong genetic determinant of the HP level in Ghanaians^26^.

In addition, associations between these polymorphisms as well as rs2000999 and serum cholesterol levels were reported^5,23,24,27–30^. Each allele of the polymorphisms that correlated with a higher HP level was associated with lower cholesterol levels despite the population, although no report is available for rs5471 yet.

Recently, human zonulin was identified as a pre-HP2 that enhances intestinal permeability by modulation of intracellular tight junctions^31^. Thus, the relationship between zonulin and the common *HP* genotypes has received attention for their potential involvement in the pathogenesis of gastrointestinal diseases and association studies with autoimmune, infective, metabolic, and tumoral diseases such as Celiac disease, obesity, and irritable bowel syndrome^32^.

Public databases provide human variation and genotype data; however, the distribution of a common *HP* polymorphism, *HP*^*del*^, and some SNPs including rs5472 and the polymorphisms responsible for 1F/S (such as rs137853233) are not available in these databases. As seen above, genetic polymorphisms of *HP* are distributed in a population-specific manner. However, to our knowledge no study so far has explored the comprehensive relationship among the polymorphisms in modern Latin Americans. In this study, to understand genetic polymorphisms in Latin American populations as the basis for an association study, we genotyped for rs5471 and rs5472, which also probably represent 1F/S, common *HP* alleles, rs2000999, and *HP*^*del*^.

## Results

### Development and validation of genotyping for rs5471 and rs5472 by real-time PCR and HRM assays

**Figure 1:**
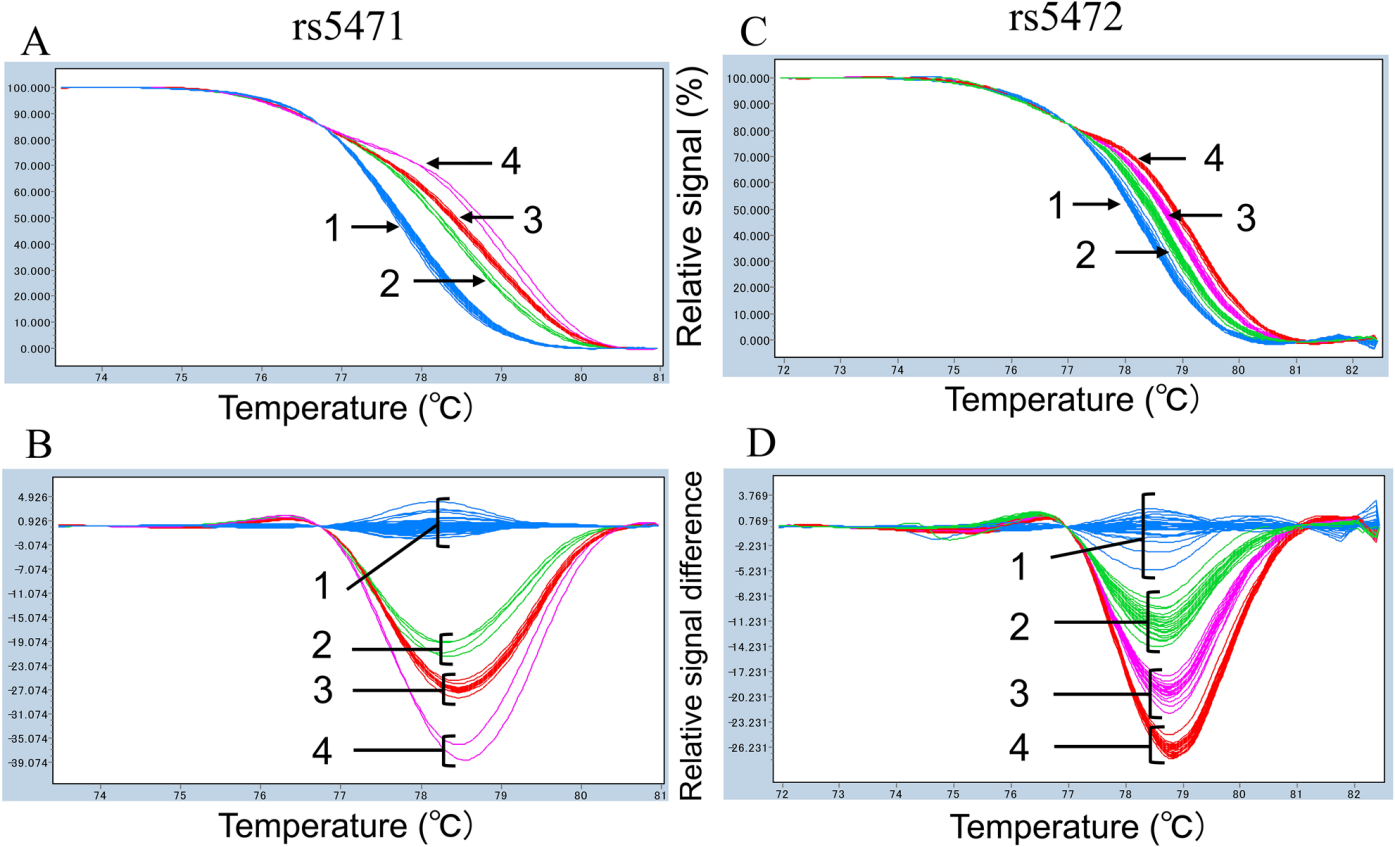
Real-time PCR and HRM analysis of *HP* promoter polymorphisms. Typical results of amplicon for rs5471 and amplicon for rs5472 on Ghanaians (n = 95) are shown. Normalized and temperature-shifted melting curves of amplicons for rs5471 (**A**) and rs5472 (**C**) and normalized and temperature-shifted difference plots for rs5471 (**B**) and rs5472 (**D**).

We developed real-time PCR and HRM assays for genotyping the *HP* promoter polymorphisms rs5471 and rs5472. As mentioned above, rs5471 is a characteristic SNP of Africans, while rs5472 is common in various populations. The frequencies of the C allele of rs5471 and G allele of rs5472 were 12.7% and 41.8%, respectively, in 122 Ghanaian subjects, whose promoter polymorphisms had already been determined by direct sequencing^33^. Because the rs5471 C allele seems to completely link with the rs5472 A allele, six haplotypes of rs5471 and rs5472 were found in Ghanaians, i.e., AA/AA, AA/CA, AG/AG, AA/AG, AG/CA, and CA/CA. Thus, in order to validate the designed HRM assays, we first examined 122 Ghanaian subjects. As a result, amplicons for rs5471 were divided into four groups, group 1 (all of 94 AA/AA, AA/AG, AG/AG), group 2 (6 of 14 AA/CA), group 3 (8 of 14 AA/CA and all 10 AG/CA), and group 4 (all of 3 CA/CA) (Fig. 1A,B). Amplicons for rs5472 were also divided into four groups, group 1 (all 45 AA/AA, AA/CA, CA/CA), group 2 (26 of 42 AA/AG), group 3 (16 of 42 AA/AG and all of 10 AG/CA), and group 4 (all of 25 AG/AG) (Fig. 1C,D). One individual had a rare base substitution at position of − 68 (rs55663121) as a heterozygote (T/C). The genotype of this individual was A/A at rs5471 and rs5472, and it belonged to group 3 of rs5471and group 1 of rs5472. In any case, we determined six rs5471 and rs5472 haplotypes accurately except one subject (heterozygote of rs55663121) when we comprehensively considered both HRM results for rs5471 and rs5472 (Table 1). We then determined the haplotypes of rs5471 and rs5472 in 416 Latin American individuals (Table 2).

**Table 1:** Haplotypes of rs5471 and rs5472 by HRM analyses.

Amplicon for rs5471	Amplicon for rs5472	Haplotype
Group 1	Group 1	AA/AA
Group 1	Group 2 or 3	AA/AG
Group 1	Group 4	AG/AG
Group 2 or 3	Group 1	AA/CA
Group 3	Group 3	AG/CA
Group 4	Group 1	CA/CA

One individual with a rare base substitution at position − 68 (rs55663121) as a heterozygote (T/C) and A/A at both SNPs shows Group 3 and Group 1 patterns for rs5471 and rs5472, respectively.

**Table 2:** Genotype distributions of three polymorphisms in several Latin American populations (n = 416).

Polymorphism	Genotype	Mexicans (n = 181)	Puerto Ricans (n = 80)	Colombians (n = 70)	Peruvians (n = 70)	Caribbeans (n = 10)	Mexican Indians (n = 5)
rs5471	A/A	180 (99.4)	77 (96.3)	69 (98.6)	70 (100)	10 (100)	5 (100)
	A/C	1 (0.6)	3 (3.8)	1 (1.4)	0	0	0
	C/C	0	0	0	0	0	0
HWE		*p* = 0.500	*p* = 0.509	*p* = 0.500			
rs5472	A/A	94 (51.9)	35 (43.8)	27 (38.6)	51 (72.9)	3 (30)	3 (60)
	A/G	70 (38.7)	33 (41.3)	41 (58.6)	18 (25.7)	4 (40)	2 (20)
	G/G	17 (9.4)	12 (15.0)	2 (2.9)	1 (1.4)	3 (30)	0
HWE		*p* = 0.417	*p* = 0.399	*p* = 0.00379	*p* = 0.809	*p* = 0.382	*p* = 0.556
Common *HP*	HP^1^/HP^1^	45 (24.9)	19 (23.8)	19 (27.1)	24 (34.3)	2 (20)	5 (100)
	HP^2^/HP^1^	93 (51.3)	35 (43.8)	30 (42.9)	34 (48.6)	8 (80)	0
	HP^2^/HP^2^	43 (23.8)	26 (32.5)	21 (30.0)	12 (17.1)	0	0
HWE		*p* = 0.711	*p* = 0.318	*p* = 0.194	*p* = 0.903	*p* = 0.127	
rs2000999	A/A	9 (5.0)	1 (1.3)	0	0	0	0
	A/G	47 (26.0)	27 (33.8)	26 (37.1)	13 (18.6)	4 (40)	0
	G/G	125 (69.1)	52 (65.0)	44 (62.9)	57 (81.4)	6 (60)	5 (100)
HWE		*p* = 0.100	*p* = 0.359	*p* = 0.0766	*p* = 0.729	*p* = 0.653	

A *p* value lower than 0.05 was interpreted as evidence that the sample was not under Hardy–Weinberg equilibrium (HWE), but after Bonferroni correction for nineteen comparisons, the statistical threshold was adjusted to 0.05/19 ≈ 0.00263.

### Allele frequency of four polymorphisms and haplotype frequency of three polymorphisms

To know more about the distribution of the *HP*^*del*^*,* which has been encountered only in East and Southeast Asia, we screened this allele and common *HP* polymorphism by TaqMan assay. However, we did not encounter any *HP*^*del*^ allele in the studied populations. The frequency of *HP*^*1*^ is known to be relatively high in Latin Americans^3,34^. We encountered it at 45.6–100% in this study, but only five samples were available from Mexican Indians (Table 2). We also genotyped rs2000999, and the results are shown in Table 2. The distributions of all polymorphisms were in Hardy–Weinberg equilibrium (HWE) in all populations except rs5472 in the Colombian population (*p* = 0.00379). However, this value was not significant after Bonferroni correction (adjusted *p* value = 0.00263, Table 2). In the studied population, five subjects had the rs5471 C allele in a heterozygous state. Whole genome DNA sequencing data of four of the five subjects having the rs5471 C allele were available in a 1,000 genome database (https://www.ncbi.nlm.nih.gov/variation/tools/1000genomes/), and the results of rs5471 obtained in this study are consistent with those of this database. On the other hand, allele frequencies of *HP*^*del*^, rs5472, and common *HP* polymorphisms were not available in this database.

### Linkage disequilibrium between three polymorphisms

Because, we found only five of the rs5471 C allele and it seemed to be associated with the A-2-G (rs5472-common-rs2000999) haplotype, we excluded this allele from further haplotype estimations. Thus, we estimated the most likely haplotypes composed of three polymorphisms, rs5472, a common *HP* polymorphism, and rs2000999 using PHASE software. The deduced haplotypes and their frequencies are shown in Table 3 together with those of Ghanaians, Mongolians, Japanese, and Europeans^12^. Previous studies suggested that rs5472 G and HP1F and rs5472 A and HP1S were in complete or almost complete linkage disequilibrium in Ghanaian, European, and Chinese populations (also see Table 4 for Ghanaians)^8,33^. Thus, as in Ghanaians (34.4%), G-1-G probably represented the 1F phenotype and is a characteristic haplotype in Europeans (17.0%). This haplotype was relatively higher (5.0–30.0%) in Latin American populations than in East Asian populations (0.1–2.8%). In addition, the frequency of A-1-G, which probably represented a 1S phenotype, was also higher in Latin American populations (28.1–80%) than in other populations (15.6–27.1%). We then calculated the linkage disequilibrium between each pair of the two polymorphisms and compared the data with those of other populations. As shown in Table 4, the common *HP* polymorphism and rs2000999 are in strong linkage disequilibrium in all populations (|D′|= 0.924–1, r^2^ = 0.101–0.375). In addition, rs2000999 is in complete linkage disequilibrium with rs5472 in Latin American populations (|D′|= 1, r^2^ = 0.250–0.615) and is similar to that of Europeans (|D′|= 1.000, r^2^ = 0.406), while the linkage disequilibrium between rs5472 and common *HP* polymorphism is much weaker than those of East Asians and is similar to that of Europeans.

**Table 3:** Comparisons of inferred haplotypes composed of three polymorphisms and their frequencies in Mongolians, Japanese, and Europeans.

	Haplotypes (rs5472–Common *HP*–rs2000999)							
	G-2-A	G-2-G	G-1-A	G-1-G	A-2-A	A-2-G	A-1-A	A-1-G
Mexicans	18.0	–	–	10.8	–	31.5*	–	39.8
Puerto Ricans	17.5	0.6	0.6	16.9	–	36.3*	–	28.1
Colombians	18.6	0.7	–	12.9	–	32.1*	–	35.7
Peruvians	9.3	–	–	5.0	–	32.1	–	53.6
Caribbeans	20.0	–	–	30.0	–	20.0	–	30.0
Mexican Indians	–	–	–	20.0	–	–	–	80.0
Ghanaians	2.5	4.9	–	34.4 (G-1F-G)	–	41.8*	0.8 (A-1S-A)	15.6 (A-1S-G)
Mongolians	22.8	0.2	0.3	2.8	0.06	50.1	–	23.9
Japanese	37.5	–	0.1	0.1	–	35.3	–	27.1
Europeans	17.5	–	0.3	17.0	–	42.0	–	23.3

1 and 2 represent *HP*^*1*^ and *HP*^*2*^, respectively. Haplotype frequencies in Japanese and Europeans are from Soejima et al.^12^, Mongolians are from Soejima et al.^22^ and unpublished results (rs5471), and Ghanaians are from Teye et al.^33^ and Soejima et al.^26^. *Haplotype containing the C allele of rs5471 seems to be included in A-2-G.

**Table 4:** Linkage disequilibrium (LD) between two polymorphisms in Latin Americans, Mongolians, Japanese, and Europeans.

Population	Coefficient	rs5472 and common *HP*	rs5472 and rs2000999	Common *HP* and rs2000999
Mexicans	|D′|	0.258	1.00	1.00
	r^2^	0.028	0.543	0.216
Puerto Ricans	|D′|	0.064	1.00	0.924
	r^2^	0.003	0.399	0.158
Colombians	|D′|	0.176	1.00	1.00
	r^2^	0.014	0.482	0.215
Peruvians	|D′|	0.402	1.00	1.00
	r^2^	0.038	0.615	0.144
Caribbeans	|D′|	0	1.00	1.00
	r^2^	0	0.250	0.375
Ghanaian	|D′|	0.641	0.570	0.508
	r^2^	0.286	0.015	0.009
Mongolians	|D′|	0.567	0.997	0.956
	r^2^	0.042	0.914	0.101
Japanese	|D′|	0.980	1.000	0.990
	r^2^	0.217	0.996	0.221
Europeans	|D′|	0.154	1.000	0.965
	r^2^	0.018	0.406	0.137

Data of Ghanaians are from Soejima et al.^26^ and Teye et al.^31^, Japanese and Europeans are from Soejima et al.^12^ and Mongolians are from Soejima et al.^22^ and unpublished results (rs5472).

### Fixation index (*F*_ST_) values among five American populations

To quantify population differences, we calculated *F*_ST_ values pairwise among the five populations (Table 5). When following the qualitative guidelines proposed by Wright^35^, there was little genetic differentiation between populations except Peruvians. On the other hand, there was a moderate differentiation between the Peruvians and Puerto Ricans or Caribbeans.

**Table 5:** *F*_ST_ values between all pairs of populations estimated from allele frequency of the *HP.*

	Mexicans	Puerto Ricans	Colombians	Peruvians	Caribbeans
Mexicans		0.0013	− 0.0037	0.0275	0.0143
Puerto Ricans			− 0.0050	0.0550	0.0027
Colombians				0.0383	0.0049
Peruvians					0.0822
Caribbeans					

## Discussion

*HP*^*del*^ homozygotes have anhaptoglobinemia, and they are at risk of suffering severe adverse effects of transfusion^9,12,36^. Thus, in the regions where *HP*^*del*^ is distributed, a genetic test to detect homozygotes before transfusion may be effective to prevent anaphylaxis. However, no study of health problems among homozygotes has been reported, and we are not able to think of any disadvantages caused by having the *HP*^*del*^ allele, except in transfusion, although HP functions as a scavenger of harmful free hemoglobin in intravascular hemolysis, and then *HP*^*del*^ homozygotes might be at a disadvantage.

In our previous study, *HP*^*del*^ was not encountered in Nepalese (Tibetans and Tamang)^13^. Tibetans and Han Chinese were reported to share relatively recent (7,000–10,000 years ago) Y and mitochondrial DNA haplotypes^37,38^. If individuals with *HP*^*del*^ had migrated to Tibet 7,000–10,000 years ago, this allele would be distributed in Tibet unless it has a deleterious effect on highlanders or it was neutral in these populations and became extinct due to genetic drift, as described in a previous study^18^. Considering these findings, the *HP*^*del*^ allele seems to have been generated relatively recently somewhere in China and spread to East and Southeast Asians in a relatively short time period. Our present result that there is no *HP*^*del*^ allele in the Latin American populations also supports this hypothesis.

Our previous studies suggested that the A-1(-G) haplotype represented the 1S phenotype and G-1(-G) represents 1F in African, European, and Chinese populations^8,33^. This means that we can predict the 1F/S subtype by genotyping rs5472. Because HP2 also contains 1F and/or 1S type sequences, it is difficult to genotype the polymorphisms directly by a conventional method. Thus previous studies, including ours, predicted 1F/S subtypes using indirect methods such as PCR to amplify the relatively large *HP*^*1*^ allele (at least 1.7 kb), followed by restriction enzyme digestion (PCR–RFLP using XbaI or DraI) to recognize a SNP that locates near and links with the polymorphisms responsible for 1F/S^7,33^. However, it is difficult to examine many samples by PCR–RFLP. A number of association studies between a common *HP* polymorphism and susceptibility to various diseases and various clinical states have been performed in previous decades^3^. Recently, high-throughput methods have been reported to impute common *HP* alleles from SNP data obtained by microarray^5,32^. As described earlier, several genetic polymorphisms of *HP* have been reported to be associated with serum HP and cholesterol levels. In addition to recently developed high-throughput methods for imputation of common *HP* alleles, the haplotype estimation of rs5472 and common *HP* polymorphisms by real-time PCR assays seemed to be useful for large-scale association studies for *HP* genotypes particularly including *HP*^*1F*^ and *HP*^*1S*^.

We observed relatively higher population differentiation statistics (*F*_ST_) between Peruvians and other Latin American populations. This may be mainly explained by the lower frequency of G-1-G (5.0% vs. 10.8–30.0%) and G-2-A (9.3% vs. 17.5–20%) haplotypes, and the higher frequency of the A-1-G (53.6% vs. 28.1–39.8%) haplotype. Although the sample size is too small (n = 5), the frequency of G-1-G was relatively lower than that of A-1-G (ratio of G-1-G/A-1-G is 0.25), and there was no *HP*^*2*^ in Mexican Indians. It is speculated that rs5471 A, rs5472 A, *HP*^*1*^, and rs2000999 G alleles and the A-1-G haplotype were prevalent, and *HP*^*2*^ and G-1-G haplotypes (this means HP 1F and mainly migrations from Europe and Africa) were rare in Native Americans. Interestingly, all of the alleles with higher frequency in the Latin Americans are associated with higher serum HP and lower cholesterol levels^5,23,24,27–30^. In addition, the genetic influx from populations of other continents into Peruvians seems to be relatively lower than into other Latin Americans, as suggested by previous studies on autosomal, X-, or Y-chromosomal and mitochondrial markers or SNPs or our *FUT2* data on the same subjects^39–41^.

The limitations of our study are as follows: (1) the sample sizes are too small to determine the precise allelic frequency of each polymorphism and to conclude that the *HP*^*del*^ is absent in these populations. (2) We could not examine the HP phenotype or serum HP concentration because only DNA samples were available. (3) We did not determine HP1F/S status of the studied samples.

## Materials and methods

This study protocol was approved by the Ethical Committee of Kurume University, Japan.

### Subjects

Genomic DNA from 122 randomly selected Ghanaians, whose promoter polymorphisms were already determined by direct sequencing analysis, was isolated as described in a previous study^33^. A total of 416 genomic DNA samples from four 1,000 Genomes project—panels (70 Puerto Ricans in Puerto Rico, MGP00004; 70 Colombians in Medellin, MGP00005; 71 of Mexican ancestry in Los Angeles, MGP00006; 70 Peruvians in Lima, MGP00011), 100 Mexican-Americans in Los Angeles, HD100MEX-2; 10 Human Variation Panel-Mexicans (mixture of seven Mexicans and three Mexican Americans), HD08; 10 Human Variation Panel-Puerto Ricans, HD09; 10 Human Variation Panel-Caribbeans. HD14; and 5 Human Variation Panel-Mexican Indians (Pima Indians from northwest Mexico), HD28 were purchased from the Coriell Institute for Medical Research (Camden, NJ, USA). Because the origin of the Caribbeans is unclear, we treated them as an independent population group. In total, we grouped the populations into six population groups (Table 2).

### Genotyping of polymorphisms

**Figure 2:**
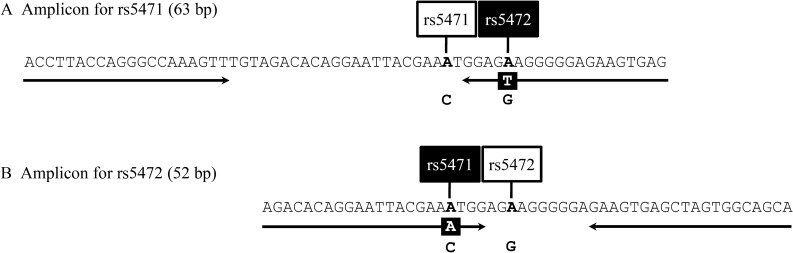
DNA sequences of amplicons for real-time PCR and HRM analyses. DNA sequences of amplicons for rs5471 (**A**) and rs5472 (**B**). The primer pairs of each amplicon are indicated by arrows. Positions and dimorphic bases of rs5471 and rs5472 are also indicated.

The zygosity of *HP*^*del*^ in addition to that of common *HP* alleles was determined using a previously described TaqMan assay^42^. Genotyping of a SNP, rs2000999, was performed as described previously^12^. Briefly, real-time PCR was carried out in 10 μl of 1 × universal probe master (FastStart, Roche Diagnostics, Tokyo, Japan) containing 0.08 μl of a predesigned TaqMan SNP genotyping assay (Assay ID C_11439045_10, ThermoFisher Scientific, Tokyo, Japan). The temperature profile was 95 °C for 10 min, followed by 45 cycles of 95 °C for 15 s and 60 °C for 45 s. Because previous studies suggested a genetic influx from Africans into modern Latin American populations, we genotyped an African-specific SNP, rs5471, in this study^8,39–41^. Real-time PCR and high-resolution melt (HRM) assays were performed for genotyping two SNPs, rs5471 and rs5472. The primer pairs and amplicons for detection of rs5471 and rs5472 polymorphisms are indicated in Fig. 2. We scanned for amplicon rs5471 of the 63 bp region, and amplicon rs5472 of the 52 bp region. Because rs5471 and rs5472 were located only 6 bp apart, we designed a reverse primer for rs5471 containing T at rs5472 and a forward primer for rs5472 containing A at rs5471 (Fig. 2). PCR amplification and HRM analysis were performed using a real-time PCR platform (LightCycler 480 instrument II, Roche Life Science) and a LightCycler 480 High Resolution Melting Master (Roche Diagnostics) as described previously^43^. Genotype frequencies were calculated by the counting method and assessed for deviations from HWE by using the exact test. Since the standard exact *p* value is overly conservative for small minor allele frequencies, we use the mid *p* value to improve this problem^44^. Maximum-likelihood haplotype frequencies were estimated using PHASE (version 2.1.1)^45^.

### Estimation of pairwise linkage disequilibrium and F_*ST*_ of genetic differentiation

Pairwise linkage disequilibrium (LD) between polymorphisms and population differentiation statistics (*F*_ST_) were calculated from the haplotype frequency data of *HP* by using the DnaSP 6.12.03 software package^46^.
